# Predictors of Newborn’s Weight for Height: A Machine Learning Study Using Nationwide Multicenter Ultrasound Data

**DOI:** 10.3390/diagnostics11071280

**Published:** 2021-07-16

**Authors:** Ki Hoon Ahn, Kwang-Sig Lee, Se Jin Lee, Sung Ok Kwon, Sunghun Na, Kyongjin Kim, Hye Sim Kang, Kyung A Lee, Hye-Sung Won, Moon Young Kim, Han Sung Hwang, Mi Hye Park, Korean Society of Ultrasound in Obstetrics Gynecology Research Group

**Affiliations:** 1 Department of Obstetrics and Gynecology, Korea University Anam Hospital, Korea University College of Medicine, Seoul 02841, Korea; 2 AI Center, Korea University Anam Hospital, Korea University College of Medicine, Seoul 02841, Korea; 3 Department of Obstetrics and Gynecology, Kangwon National University Hospital, Kangwon National University School of Medicine, Kangwon, Chuncheon 24289, Korea; 4 Department of Preventive Medicine, Kangwon National University School of Medicine, Kangwon, Chuncheon 24289, Korea; 5 Department of Obstetrics and Gynecology, Presbyterian Medical Center, Jeonju 54987, Korea; 6 Department of Obstetrics and Gynecology, Jeju National University, Jeju 63241, Korea; 7 Department of Obstetrics and Gynecology, Ewha Medical Center, Ewha Medical Institute, Ewha Womans University College of Medicine, Seoul 07804, Korea; 8 Department of Obstetrics and Gynecology, Asan Medical Center, University of Ulsan College of Medicine, Seoul 05505, Korea; 9 Department of Obstetrics and Gynecology, CHA Gangnam Medical Center, CHA University, Seoul 06135, Korea; 10 Department of Obstetrics and Gynecology, Research Institute of Medical Science, Konkuk University School of Medicine, Seoul 05030, Korea

**Keywords:** newborn, weight, height, estimated fetal weight, abdominal circumference

## Abstract

There has been no machine learning study with a rich collection of clinical, sonographic markers to compare the performance measures for a variety of newborns’ weight-for-height indicators. This study compared the performance measures for a variety of newborns’ weight-for-height indicators based on machine learning, ultrasonographic data and maternal/delivery information. The source of data for this study was a multi-center retrospective study with 2949 mother–newborn pairs. The mean-squared-error-over-variance measures of five machine learning approaches were compared for newborn’s weight, newborn’s weight/height, newborn’s weight/height^2^ and newborn’s weight/hieght^3^. Random forest variable importance, the influence of a variable over average node impurity, was used to identify major predictors of these newborns’ weight-for-height indicators among ultrasonographic data and maternal/delivery information. Regarding ultrasonographic fetal biometry, newborn’s weight, newborn’s weight/height and newborn’s weight/height^2^ were better indicators with smaller mean-squared-error-over-variance measures than newborn’s weight/height^3^. Based on random forest variable importance, the top six predictors of newborn’s weight were the same as those of newborn’s weight/height and those of newborn’s weight/height^2^: gestational age at delivery time, the first estimated fetal weight and abdominal circumference in week 36 or later, maternal weight and body mass index at delivery time, and the first biparietal diameter in week 36 or later. These six predictors also ranked within the top seven for large-for-gestational-age and the top eight for small-for-gestational-age. In conclusion, newborn’s weight, newborn’s weight/height and newborn’s weight/height^2^ are more suitable for ultrasonographic fetal biometry with smaller mean-squared-error-over-variance measures than newborn’s weight/height^3^. Machine learning with ultrasonographic data would be an effective noninvasive approach for predicting newborn’s weight, weight/height and weight/height^2^.

## 1. Introduction

Newborns’ underweight and children’s obesity are significant contributors for disease burden on the globe. One in every seven newborns in the world suffered from underweight in 2015 and these babies are more likely to experience death in the initial 28 days of life than common babies [1]. Similarly, 40 million children aged five or less in the world were characterized by overweight or obesity in 2016 [2], and this is likely to cause various diseases in their subsequent life such as asthma, cardiovascular disorders, depression, diabetes, dyslipidemia and hypertension [3,4,5,6,7,8]. In this context, the World Health Organization champions a global goal “No Increase in Childhood Overweight by 2025” [9].

Likewise, existing literature has attempted to examine newborn’s weight and its significant predictor variables among ultrasonographic data and maternal/delivery information [10,11,12,13]. These studies adopted linear regression, and hence they could not analyze (1) which predictor variables are more important for predicting newborn’s weight, or (2) what time is the best for taking ultrasonographic data. To overcome these limitations, a more recent study employed machine learning and made predictions for newborn’s body mass index from ultrasonographic data and maternal/delivery information [14]. The findings of this study agreed with those of existing literature stating that newborn’s weight/height^2^ would be a good alternative measure of newborn’s adiposity to newborn’s weight [15,16,17].

However, an optimal index for classifying underweight and overweight in children under 2 years of age has not been established yet, while conventional studies still ignore newborn’s weight/height and weight/height^3^ (Ponderal Index). Here, the Ponderal Index is designed to reflect the three-dimensional (volume) information (height^3^) [18]. To our best knowledge, there has been no machine learning study with a rich collection of clinical sonographic markers to compare the performance measures for a variety of newborns’ weight-for-height indicators. In this context, this study compared the performance measures for a variety of newborn’s weight-for-height indicators based on machine learning, ultrasonographic data and maternal/delivery information. This study includes four weight-for-height indicators, that is, newborn’s weight, weight/height, weight/height^2^ and weight/height^3^. In addition, this study features 64 clinical, sonographic markers and 2949 mother–baby pairs. The ultimate goal of this study is to test the following null and alternative hypotheses:

**Null Hypothesis:** Newborn’s weight, newborn’s weight/height, newborn’s weight/height^2^ and newborn’s weight/height^3^ are equally suitable for ultrasonographic fetal biometry.

**Alternative Hypothesis:** Newborn’s weight, newborn’s weight/height, newborn’s weight/height^2^ and newborn’s weight/height^3^ are not equally suitable for ultrasonographic fetal biometry.

## 2. Materials and Methods

### 2.1. Participants

The source of data for this multi-center retrospective study was the same as in [14], the medical records of 2949 mother–baby pairs (see [14] for more detailed description). The study period was September 2019–March 2021 and the participating institutions were 48 general hospitals. This study was approved by institutional review boards of the forty-eight hospitals such as Korea University Anam Hospital (2019AN0433) participating in the study. Informed consent was waived by the institutional review boards. No administrative permissions or licenses were acquired by the authors to access the data used in this study. Then, data collection, analysis and interpretation followed.

### 2.2. Variables

The dependent variables were newborn’s weight, weight/height and weight/height^3^. Newborn’s weight and height were recorded at the time of birth. The following 64 independent variables were considered: (1) maternal data including age (years), children alive, height, pre-gestational weight, weight at delivery time, pre-gestational body mass index, body mass index at delivery time, term births, preterm births, abortions; (2) gestational age, ultrasound measures (see their notations in Table S1 (Supplementary Materials)); and (3) delivery/newborn data such as gestational age at delivery (weeks/days), Apgar scores in 1 and 5 min after delivery, caesarean delivery methods (no vs. yes), newborn’s sex—female (no vs. yes), neonatal intensive care unit hospitalization (no vs. yes). All participating institutions adopted Hadlock’s formula [19] for the estimation of EFW (except one participating institution that employed the Shinozuka’s formula [20]). These formulas use the same parameters and register similar performances to predict newborn’s weight [21].

### 2.3. Analysis

Five machine learning approaches were adopted for the prediction of newborn’s weight, weight/height and weight/height^3^: linear regression, random forest and artificial neural networks with one, two and three hidden layers [14,22]. Data on 2949 mother–baby pairs were split into training and validation sets with a 75:25 ratio (2212 vs. 737 mother–baby pairs). The mean squared error (MSE), the average of the squares of errors among 737 mother–baby pairs, was employed as a performance measure. The unit of the MSE is the squared unit of the dependent variable. The MSE is not appropriate for the comparison of model performance across different dependent variables with different units. For this reason, the MSE divided by the variance of the dependent variable (MSE over variance) was introduced for the comparison of model performance across different dependent variables with different units. Finally, random forest variable importance, the influence of a variable over average node impurity, was introduced for identifying most important predictor variables of newborn’s weight, weight/height and weight/height^3^ among ultrasonographic data and maternal/delivery information. R-Studio was used for the analysis on March 2021. It needs to be noted that the results for newborn’s weight/height^2^ were adopted from [14] and were compared with those for newborn’s weight, weight/height and weight/height^3^ in this study.

## 3. Results

Descriptive statistics in this study are given in Table 1. The respective median (Q2) values of newborn’s weight, weight/height, weight/height^3^, GA36AC1 (the first abdominal circumference in week 36 or later), GA36EFW1 (the first estimated fetal weight in week 36 or later) and gestational age at delivery time were 3.17 kg, 6.36 kg/m, 25.68 kg/m^3^, 322 mm, 2866 g and 38 weeks. The respective median values of GA21AC1 (the first abdominal circumference during week 21–week 35) and maternal body mass index at delivery time were 214.70 mm and 26.04 kg/m^2^. The proportion of neonatal intensive care unit hospitalization was 12% (354/2949). The MSEs of the five machine learning models for newborn’s weight, weight/height, weight/height^2^ and weight/height^3^ are presented in Table 2. The data were split, and the analysis was performed three times; then, the average MSE was obtained for each of the five statistical methods. Linear regression and the random forest were better models with smaller MSEs than the artificial neural networks for predicting newborn’s weight-for-height indicators. More importantly, newborn’s weight, newborn’s weight/height and newborn’s weight/height^2^ were better indicators with smaller MSE-over-variance measures than newborn’s weight/height^3^.

Based on random forest variable importance, the top six predictor variables of newborn’s weight were the same with those of newborn’s weight/height and newborn’s weight/height^2^: Gestational age at delivery time, the first EFW and AC in week 36 or later, maternal weight and body mass index at delivery time, and the first BPD (biparietal diameter) in week 36 or later (See Table 3, Table 4, Table 5, Table S2(1–3) (Supplementary Materials) and Figure S1(1–3) (Supplementary Materials) in this study, Table 3 and Figure 1 in [14]). Eight among the top ten predictor variables of newborn’s weight/height^3^ were identical to those of newborn’s weight, weight/height and weight/height^2^. However, the importance ranking of the first EFW in week 36 or later was lower for newborn’s weight/height^3^ than for the other three indicators, and vice versa for the first AC during week 21–week 35. Indeed, the results of linear regression are informative regarding the effects of important predictor variables on newborn’s weight or weight/height. For example, newborn’s weight will increase by 170 g if gestational age at delivery time increases by 1 week. Newborn’s weight/height will increase by 0.05 g/m if the first EFW in week 36 or later increases by 1 g.

**Figure 1:**
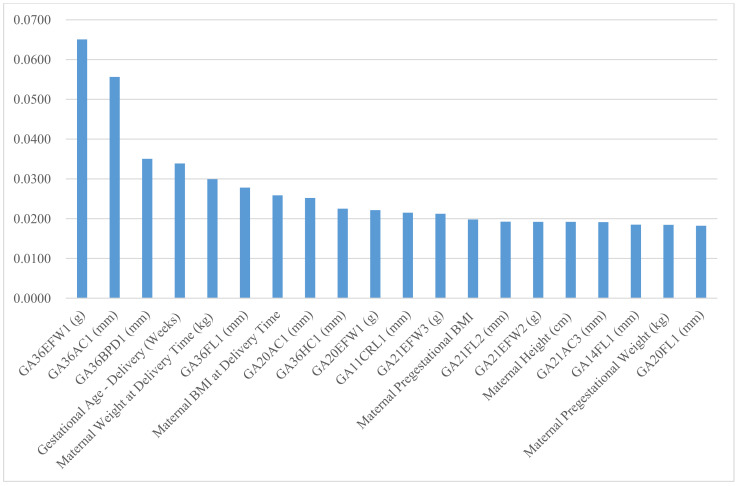
Random forest variable importance values of top 20 predictors for newborn’s large-for-gestational-age.

**Figure 2:**
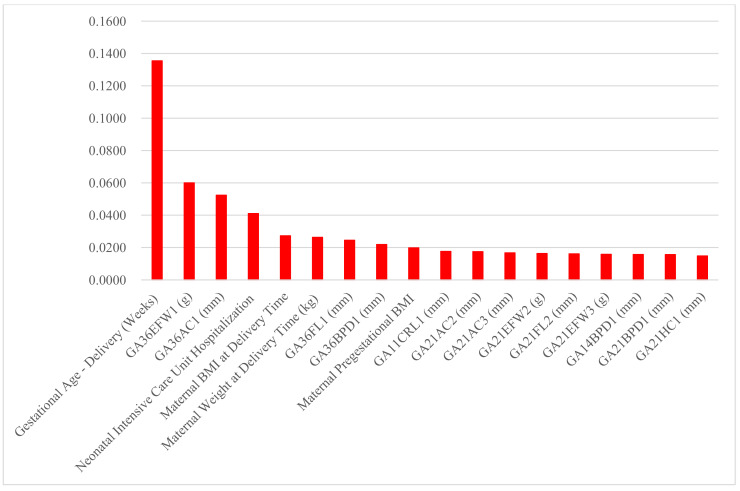
Random forest variable importance values of top 20 predictors for newborn’s small-for-gestational-age.

Finally, the random forest variable importance of predictors for large-for-gestational-age (LGA) and small-for-gestational-age (SGA) are presented in Figure 1 and Figure 2, respectively. The top six predictor variables of newborn’s weight, weight/height and weight/height^2^ also ranked within the top seven for LGA and the top eight for SGA: gestational age at delivery time, the first EFW and AC in week 36 or later, maternal weight and body mass index at delivery time, and the first BPD in week 36 or later. Moreover, the importance rankings of the top three predictors for newborn’s weight, weight/height and weight/height^2^ were within the top four for LGA and the top three for SGA as well: gestational age at delivery time, and the first EFW and AC in week 36 or later.

The results of this study support the alternative hypothesis: newborn’s weight, newborn’s weight/height, newborn’s weight/height^2^ and newborn’s weight/height^3^ are not equally suitable for ultrasonographic fetal biometry. It was found in this study that newborn’s weight, newborn’s weight/height and newborn’s weight/height^2^ are more suitable for ultrasonographic fetal biometry than newborn’s weight/height^3^.

## 4. Discussion

### 4.1. Principal Findings

Newborn’s weight/height and newborn’s weight/height^2^ are more suitable for ultrasonographic fetal biometry with smaller MSE-over-variance measures than newborn’s weight/height^3^. The top six predictor variables of newborn weight were the same as those of newborn weight/height and those of newborn weight/height^2^: gestational age at delivery time, the first EFW and AC in week 36 or later, maternal weight and body mass index at delivery time, and the first BPD in week 36 or later. These six predictors also ranked within the top seven for large-for-gestational-age and the top eight for small-for-gestational-age.

### 4.2. Clinical and Research Implications

The findings of this study above are consistent with those of the previous study [14]: week 36 or later is the best time to take ultrasonographic data, and AC and EFW are the most important predictor variables of newborn’s weight/height^2^ together with gestational age at delivery and maternal body mass index at delivery. However, the previous study ignored newborn’s weight/height, which can be another good alternative measure of newborn’s adiposity to newborn’s weight. As a matter of fact, there is no consensus on the best weight-for-height indicator for newborns and children under the age of 2, in part because babies born earlier are more heterogeneous in terms of weight for height than babies born later [18]. Newborn thinness is considered to be a risk factor for adult chronic disease, but it is not clear which of a newborn’s weight-for-height indicators (e.g., weight/height, weight/height^2^, weight/height^3^) are the best indicators for adult chronic disease [18]. Given that newborn thinness is known to be a risk factor for adult chronic disease, it would be worthwhile to shift our attention to newborn weight-for-height indicators and their prenatal predictors. This will help to develop a new research tradition covering health conditions across different life periods, i.e., prenatal, newborns, children and adults. In this context, this study compared the performance measures for a variety of newborn weight-for-height indicators based on machine learning, ultrasonographic data and maternal/delivery information. To the best of our knowledge, there has been no study on this topic in this direction. The findings of this study suggest that machine learning with ultrasonographic data would be an effective noninvasive approach for predicting a newborn’s weight, weight/height and weight/height^2^. Specifically, the results of this study bring the following clinical implication for the prognosis of adiposity for newborns and children under the age of 2 (with no current consensus on their best weight-for-height indicators): clinicians are recommended to use a newborn’s weight, weight/height or weight/height^2^ as an indicator of a newborn’s adiposity when they employ ultrasonographic fetal biometry.

### 4.3. Strengths and Limitations

To the best of our knowledge, there has been no machine learning study with a rich collection of clinical, sonographic markers to compare the performance measures for a variety of newborns’ weight-for-height indicators. In this context, this study compared the performance measures for a variety of newborn’s weight-for-height indicators based on machine learning, ultrasonographic data and maternal/delivery information. This study included four weight-for-height indicators, that is, newborn’s weight, weight/height, weight/height^2^ and weight/height^3^. In addition, this study featured 64 clinical, sonographic markers and 2949 mother–baby pairs. However, this study had some limitations. Firstly, this study did not include possible mediating effects. Secondly, this study did not consider socioeconomic determinants, disease information (diabetes, gastroesophageal reflux disease, hypertension, periodontitis), medication history (benzodiazepine, calcium channel blocker, nitrate, progesterone, proton pump inhibitor, sleeping pills, antidepressant) and obstetric information (in vitro fertilization, myoma uteri, prior cone). These factors have been reported to influence delivery outcome [23,24,25] and it would be a useful extension to consider these new variables. Thirdly, additional examination of symptomatic vs. asymptomatic, single vs. multiple gestation, is expected to provide more insights and implications on this important topic.

## 5. Conclusions

This is the first study to compare the performance measures for a variety of newborn’s weight-for-height indicators based on machine learning, ultrasonographic data and maternal/delivery information. Newborn’s weight, newborn’s weight/height and newborn’s weight/height^2^ are more suitable for ultrasonographic fetal biometry with smaller MSE-over-variance measures than newborn’s weight/height^3^. Machine learning with ultrasonographic data would be an effective noninvasive approach for predicting newborn’s weight, weight/height and weight/height^2^.

## Figures and Tables

**Table 1:** Descriptive statistics.

Continuous Variable	*SD*	*Min*	*Q1*	*Median*	*Q3*	*Max*
Newborn’s Weight (kg)	0.42	1.58	2.92	3.17	3.44	4.67
Newborn’s Weight/Height (kg/m)	0.73	3.66	5.92	6.36	6.82	9.16
Newborn’s Weight/Height^3^ (kg/m^3^)	3.06	14.64	23.90	25.68	27.60	37.04
Maternal Age	4.01	19.00	31.00	33.00	36.00	48.00
Maternal Height (cm)	5.17	140.00	158.00	161.00	165.00	181.00
Maternal Pregestational Weight (kg)	8.11	34.00	51.00	55.00	60.00	99.00
Maternal Weight at Delivery Time (kg)	8.60	45.00	62.85	68.00	74.00	92.80
Maternal Pregestational BMI	3.11	14.50	19.49	21.05	23.23	39.86
Maternal BMI at Delivery Time	3.11	16.33	24.21	26.04	28.23	40.00
GA11CRL1 (mm)	8.66	32.60	50.00	56.00	61.40	79.80
GA11NT1 (mm)	1.19	0.04	1.00	1.20	1.50	40.00
GA14BPD1 (mm)	3.68	23.10	32.40	34.70	36.40	67.00
GA14HC1 (mm)	10.29	72.60	123.70	126.40	128.70	200.00
GA14AC1 (mm)	11.58	34.00	101.40	107.30	112.00	219.00
GA14FL1 (mm)	2.96	9.10	18.00	19.80	21.30	32.50
GA14EFW1 (g)	31.88	14.00	137.00	152.00	165.00	345.00
GA20BPD1 (mm)	4.57	38.00	48.70	51.40	54.00	67.70
GA20HC1 (mm)	14.27	118.40	182.10	191.20	195.60	250.50
GA20AC1 (mm)	42.18	108.70	157.00	166.70	175.00	2113.30
GA20FL1 (mm)	3.67	25.90	32.80	35.00	37.30	45.50
GA20EFW1 (g)	110.31	109.00	367.00	425.00	481.00	980.00
GA21BPD1 (mm)	6.62	46.70	61.45	65.80	70.50	85.00
GA21HC1 (mm)	22.76	169.70	233.50	244.20	249.10	839.00
GA21AC1 (mm)	24.22	108.30	200.50	214.70	231.60	310.50
GA21FL1 (mm)	5.16	32.40	43.90	47.00	51.00	62.40
GA21EFW1 (g)	302.32	177.00	731.00	868.00	1098.00	2185.00
GA21BPD2 (mm)	5.57	61.00	73.60	77.40	80.60	92.90
GA21HC2 (mm)	15.30	193.20	276.90	283.00	284.60	386.20
GA21AC2 (mm)	23.64	155.00	244.00	258.40	272.10	369.70
GA21FL2 (mm)	4.58	43.40	53.40	56.40	59.20	68.60
GA21EFW2 (g)	393.44	644.00	1293.00	1508.00	1747.00	3569.00
GA21BPD3 (mm)	3.71	74.50	82.40	85.00	86.20	93.60
GA21HC3 (mm)	10.46	201.30	306.20	307.00	307.00	390.60
GA21AC3 (mm)	17.49	227.00	280.90	293.00	297.60	381.40
GA21FL3 (mm)	3.10	55.00	61.00	63.20	64.00	69.70
GA21EFW3 (g)	322.77	1211.00	1953.00	2186.00	2273.00	3661.00
GA36BPD1 (mm)	3.29	75.10	89.00	90.80	92.60	103.40
GA36HC1 (mm)	9.36	206.00	323.10	324.70	326.00	419.90
GA36AC1 (mm)	15.32	243.60	314.00	322.00	330.50	460.10
GA36FL1 (mm)	2.79	56.00	67.00	68.90	70.30	89.00
GA36EFW1 (g)	299.59	1577.00	2706.00	2866.00	3036.00	4172.00
Gestational Age—Delivery (Weeks)	1.53	20.00	38.00	38.00	39.00	42.00
Apgar Score in 1 Minute After Delivery	0.50	0.00	8.00	8.00	9.00	20.00
Apgar Score in 5 Minutes After Delivery	0.50	0.00	9.00	9.00	10.00	10.00

Notes: *SD:* Standard Deviation; *AC:* Abdominal Circumference (mm); *BPD:* Biparietal Diameter (mm); *CRL:* Crown-Rump Length (mm); *EFW:* Estimated Fetal Weight (g); *FL:* Femur Length (mm); *HC:* Head Circumference (mm); *NT:* Nuchal Translucency (mm); *GA11:* Gestational Age, Week 11–Week 13; *GA14:* Gestational Age, Week 14–Week 19; *GA20:* Gestational Age, Week 20; *GA21:* Gestational Age, Week 21–Week 35; *GA36:* Gestational Age, Week 36 or Later.

**Table 2:** Model performance: average mean squared error.

Dependent Variable/Model	*Run 1*	*Run 2*	*Run 3*	*MSE*	*MSE/V* ^†^
					
*Newborn’s Weight*					
Linear Regression	0.0579	0.1151	0.0584	0.0772	0.4376
Random Forest	0.0716	0.1236	0.0776	0.0909	0.5153
ANN 1 Layer *	0.2152	0.2866	0.3180	0.2733	1.5493
ANN 2 Layers	0.1748	0.5565	0.0858	0.2724	1.5442
ANN 3 Layers	0.2277	0.3174	0.2260	0.2571	1.4575
					
*Newborn’s Weight/Height*					
Linear Regression	0.2593	0.5336	0.2656	0.3528	0.6620
Random Forest	0.2934	0.5583	0.3130	0.3882	0.7285
ANN 1 Layer	32.4925	32.9507	32.7599	32.7344	61.4269
ANN 2 Layers	32.4933	33.5946	33.0660	33.0513	62.0216
ANN 3 Layers	32.5195	33.5437	32.7257	32.9296	61.7932
					
*Newborn’s Weight/Height^3^*					
Linear Regression	8.7276	23.9906	9.5329	14.0837	1.5041
Random Forest	8.9470	23.0724	9.3411	13.7868	1.4724
ANN 1 Layer	650.1868	668.5456	656.0115	658.2480	70.2986
ANN 2 Layers	652.3180	661.5827	648.1057	654.0021	69.8452
ANN 3 Layers	659.6003	656.9730	659.0836	658.5523	70.3311
					
*Newborn’s Weight/Height^2^* [14]					
Linear Regression	1.7933	1.9526	2.4774	2.0744	0.8747
Random Forest	1.8359	2.0782	2.5688	2.1610	0.9112
ANN 1 Layer	140.0307	158.5399	153.5595	150.7100	63.5478
ANN 2 Layers	140.0916	158.5026	165.5652	154.7198	65.2386
ANN 3 Layers	139.3295	158.6813	159.7421	152.5843	64.3381

Note: * ANN Artificial Neural Network, ^†^ MSE/V Average Mean Squared Error/Variance.

**Table 3:** Random forest variable importance (VI) and regression coefficient from run 1: top 40 variables (dependent variable: newborn’s weight [g]).

Variable	Random Forest		Linear Regression	
	*VI Value*	*VI Rank*	*Coefficient*	*p-Value*
Gestational Age—Delivery (Weeks)	81437955	1	* 170.2000	0.0000
GA36EFW1 (g)	59566065	2	* 0.3300	0.0000
GA36AC1 (mm)	40359557	3	* 3.3950	0.0055
Maternal Weight at Delivery Time (kg)	12600886	4	−2.9740	0.8606
GA36BPD1 (mm)	11536549	5	3.5670	0.2456
Maternal BMI at Delivery Time	9183122	6	25.9200	0.5537
Neonatal Intensive Care Unit Hospitalization	8666421	7	* −45.8000	0.0265
GA36FL1 (mm)	8167498	8	0.8415	0.8153
GA11CRL1 (mm)	7670171	9	−0.1804	0.8833
GA21AC1 (mm)	7394983	10	1.7720	0.0624
GA21BPD2 (mm)	7337688	11	3.0080	0.3310
Maternal Pregestational BMI	6918490	12	−12.7300	0.7878
GA21AC2 (mm)	6435064	13	1.2240	0.1581
GA21AC3 (mm)	6019105	14	* 3.7860	0.0039
GA36HC1 (mm)	5840288	15	−0.1518	0.8389
GA21BPD1 (mm)	5804584	16	3.5090	0.2108
GA20AC1 (mm)	5174051	17	0.2379	0.3931
GA20EFW1 (g)	5113174	18	−0.2566	0.1524
Maternal Pregestational Weight (kg)	5066332	19	1.8500	0.9193
GA21EFW1 (g)	4834716	20	* 0.2289	0.0472
GA21FL2 (mm)	4749409	21	−3.1040	0.4481
GA20HC1 (mm)	4713837	22	−0.0199	0.9811
GA21HC2 (mm)	4646094	23	−0.6821	0.3531
Apgar Score in 1 Minute After Delivery	4645375	24	0.4369	0.9574
Apgar Score in 5 Minutes After Delivery	4632233	25	2.7750	0.8193
GA21EFW3 (g)	4602151	26	0.0566	0.5850
Maternal Height (cm)	4570952	27	8.3310	0.3864
GA14BPD1 (mm)	4472051	28	* 6.8380	0.0270
Maternal Age	4345422	29	−2.3960	0.1433
GA20BPD1 (mm)	4297216	30	−6.3500	0.0544
GA21EFW2 (g)	4291562	31	* 0.2961	0.0005
GA21FL1 (mm)	4270426	32	−4.0100	0.2977
GA21HC1 (mm)	4261178	33	−0.3190	0.3783
GA20FL1 (mm)	4222331	34	* 9.9590	0.0214
GA14FL1 (mm)	4186594	35	* −9.4160	0.0486
GA11NT1 (mm)	4115745	36	−2.3880	0.6990
GA14AC1 (mm)	3751675	37	−0.6944	0.5587
GA14HC1 (mm)	3564844	38	0.9472	0.3295
GA21D2	3276785	39	* −15.1600	0.0002
GA36W1	3209062	40	* −90.3100	0.0000

Notes: * p-Value < 0.05; *AC:* Abdominal Circumference (mm); *BPD:* Biparietal Diameter (mm); *CRL:* Crown-Rump Length (mm); *EFW:* Estimated Fetal Weight (g); *FL:* Femur Length (mm); *HC:* Head Circumference (mm); *NT:* Nuchal Translucency (mm); *GA11:* Gestational Age, Week 11–Week 13; *GA14:* Gestational Age, Week 14–Week 19; *GA20:* Gestational Age, Week 20; *GA21:* Gestational Age, Week 21–Week 35; *GA36:* Gestational Age, Week 36 or Later; *W/D:* Gestational Age–Weeks/Days.

**Table 4:** Random forest variable importance (VI) and regression coefficient from run 1: top 40 variables (dependent variable: benn index: newborn’s weight/height).

Variable	Random Forest		Linear Regression	
	*VI Value*	*VI Rank*	*Coefficient*	*p-Value*
Gestational Age—Delivery (Weeks)	213	1	* 0.2775	0.0000
GA36EFW1 (g)	162	2	* 0.0005	0.0026
GA36AC1 (mm)	138	3	* 0.0070	0.0050
Maternal Weight at Delivery Time (kg)	38	4	0.0093	0.7880
Maternal BMI at Delivery Time	37	5	0.0004	0.9967
GA36BPD1 (mm)	36	6	0.0072	0.2503
GA21AC1 (mm)	32	7	0.0033	0.0855
GA11CRL1 (mm)	32	8	−0.0002	0.9216
GA21BPD2 (mm)	31	9	0.0041	0.5133
GA21AC2 (mm)	27	10	0.0014	0.4243
GA36FL1 (mm)	23	11	0.0039	0.5915
Maternal Pregestational BMI	22	12	−0.0324	0.7362
GA21EFW2 (g)	22	13	* 0.0007	0.0001
Maternal Age	22	14	−0.0049	0.1416
GA21AC3 (mm)	21	15	* 0.0061	0.0218
GA21EFW1 (g)	20	16	* 0.0006	0.0166
GA21HC2 (mm)	20	17	−0.0010	0.5015
Neonatal Intensive Care Unit Hospitalization	20	18	−0.0460	0.2719
GA21BPD1 (mm)	19	19	0.0014	0.8032
GA36HC1 (mm)	18	20	−0.0016	0.2824
GA20AC1 (mm)	18	21	0.0007	0.2050
Maternal Pregestational Weight (kg)	18	22	0.0095	0.7981
GA14FL1 (mm)	18	23	−0.0176	0.0694
Apgar Score in 1 Minute After Delivery	17	24	0.0016	0.9248
GA21FL1 (mm)	17	25	* −0.0188	0.0166
GA21EFW3 (g)	17	26	0.0001	0.5429
GA21HC1 (mm)	17	27	−0.0008	0.2818
GA20EFW1 (g)	17	28	−0.0006	0.0912
Maternal Height (cm)	17	29	−0.0026	0.8923
GA21FL2 (mm)	16	30	−0.0125	0.1315
GA20BPD1 (mm)	16	31	−0.0076	0.2596
GA14BPD1 (mm)	16	32	* 0.0145	0.0214
GA20HC1 (mm)	15	33	−0.0004	0.8220
GA11W1	14	34	0.0061	0.8621
GA20FL1 (mm)	14	35	0.0134	0.1265
GA11NT1 (mm)	13	36	−0.0048	0.7014
Apgar Score in 5 Minutes After Delivery	13	37	0.0130	0.5985
GA14AC1 (mm)	13	38	−0.0015	0.5294
GA14HC1 (mm)	12	39	0.0002	0.9016
GA14EFW1 (g)	12	40	0.0004	0.7231

Notes: * p-Value < 0.05; *AC:* Abdominal Circumference (mm); *BPD:* Biparietal Diameter (mm); *CRL:* Crown-Rump Length (mm); *EFW:* Estimated Fetal Weight (g); *FL:* Femur Length (mm); *HC:* Head Circumference (mm); *NT:* Nuchal Translucency (mm); *GA11:* Gestational Age, Week 11–Week 13; *GA14:* Gestational Age, Week 14–Week 19; *GA20:* Gestational Age, Week 20; *GA21:* Gestational Age, Week 21–Week 35; *GA36:* Gestational Age, Week 36 or Later; *W/D:* Gestational Age–Weeks/Days.

**Table 5:** Random forest variable importance (VI) and regression coefficient from run 1: top 40 variables (dependent variable: ponderal index: newborn’s weight/height^3^).

Variable	Random Forest		Linear Regression	
	*VI Value*	*VI Rank*	*Coefficient*	*p-Value*
GA21AC1 (mm)	1804	1	0.0061	0.6314
GA36AC1 (mm)	1598	2	0.0296	0.0697
Gestational Age—Delivery (Weeks)	1417	3	* 0.3250	0.0000
GA21BPD2 (mm)	1230	4	0.0145	0.7260
Maternal BMI at Delivery Time	1205	5	−0.1966	0.7364
GA36EFW1 (g)	1196	6	0.0006	0.5464
GA21AC2 (mm)	1068	7	−0.0012	0.9168
Maternal Age	1054	8	−0.0311	0.1542
GA11CRL1 (mm)	868	9	0.0031	0.8499
GA21EFW2 (g)	808	10	* 0.0031	0.0064
Maternal Pregestational BMI	728	11	−0.2214	0.7256
Apgar Score in 5 Minutes After Delivery	727	12	−0.1616	0.3187
GA21EFW1 (g)	719	13	* 0.0032	0.0373
GA36BPD1 (mm)	672	14	0.0163	0.6903
GA21HC2 (mm)	671	15	−0.0012	0.9037
Maternal Weight at Delivery Time (kg)	662	16	0.0748	0.7408
GA21FL2 (mm)	657	17	* −0.1148	0.0356
GA20FL1 (mm)	572	18	−0.0284	0.6232
GA20BPD1 (mm)	559	19	0.0174	0.6932
GA20EFW1 (g)	558	20	−0.0032	0.1774
Maternal Height (cm)	545	21	−0.1067	0.4059
GA36FL1 (mm)	541	22	0.0587	0.2220
GA11NT1 (mm)	530	23	−0.0205	0.8034
GA21BPD1 (mm)	527	24	−0.0365	0.3298
GA14BPD1 (mm)	520	25	0.0536	0.1934
GA21FL1 (mm)	512	26	* −0.1944	0.0002
GA20AC1 (mm)	489	27	0.0057	0.1269
GA20HC1 (mm)	472	28	−0.0023	0.8358
Maternal Pregestational Weight (kg)	462	29	0.0990	0.6846
GA21AC3 (mm)	462	30	0.0126	0.4697
GA14HC1 (mm)	444	31	−0.0049	0.7033
GA14FL1 (mm)	437	32	−0.0740	0.2455
Apgar Score in 1 Minute After Delivery	431	33	0.0915	0.4015
GA11W1	426	34	−0.0138	0.9522
GA21FL3 (mm)	407	35	0.0151	0.8092
GA21HC1 (mm)	398	36	−0.0063	0.1907
GA21BPD3 (mm)	375	37	−0.0044	0.9290
GA21EFW3 (g)	374	38	0.0005	0.7374
GA14AC1 (mm)	370	39	−0.0016	0.9180
GA36HC1 (mm)	355	40	−0.0150	0.1332

Notes: * p-Value < 0.05; *AC:* Abdominal Circumference (mm); *BPD:* Biparietal Diameter (mm); *CRL:* Crown-Rump Length (mm); *EFW:* Estimated Fetal Weight (g); *FL:* Femur Length (mm); *HC:* Head Circumference (mm); *NT:* Nuchal Translucency (mm); *GA11:* Gestational Age, Week 11–Week 13; *GA14:* Gestational Age, Week 14–Week 19; *GA20:* Gestational Age, Week 20; *GA21:* Gestational Age, Week 21–Week 35; *GA36:* Gestational Age, Week 36 or Later; *W/D:* Gestational Age–Weeks/Days.

## Data Availability

The datasets used and/or analyzed during the current study are available from the corresponding authors on reasonable request.

## Supplementary Materials

The following are available online at https://www.mdpi.com/article/10.3390/diagnostics11071280/s1, Table S1: Notations of Gestational Age and Ultrasound Measures, Table S2: Random Forest Variable Importance (VI) and Regression Coefficient from Run 1: All Variables (S2-1 Newborn’s Weight, S2-2 Newborn’s Weight/Height, S2-3 Newborn’s Weight/Height^3^), Figure S1: Random Forest Variable Importance Values of Top 20 Predictors (S1-1 Newborn’s Weight, S1-2 Newborn’s Weight/Height, S1-3 Newborn’s Weight/Height^3^).
