# Supplementary material for: Predictors of Newborn’s Weight for Height: A Machine Learning Study Using Nationwide Multicenter Ultrasound Data
Source: Diagnostics (Basel). 2021 Jul 16;11(7):1280. doi: 10.3390/diagnostics11071280 (PMC8304217; doi:10.3390/diagnostics11071280)

Table S1 Notations of Gestational Age and Ultrasound Measures

	Week 11-13	*Week 14-19*	*Week 20*	*Week 21-35*			*Week 36-*
**Gestational Age (Weeks)**	GA11W1	GA14W1	GA20W1	GA21W1	GA21W2	GA21W3	GA36W1
**Gestational Age (Days)**	GA11D1	GA14D1	GA20D1	GA21D1	GA21D2	GA21D3	GA36D1
**Crown-Rump Length**	GA11CRL1						
**Nuchal Translucency**	GA11NT1						
**Biparietal Diameter**		GA14BPD1	GA20BPD1	GA21BPD1	GA21BPD2	GA21BPD3	GA36BPD1
**Head Circumference**		GA14HC1	GA20HC1	GA21HC1	GA21HC2	GA21HC3	GA36HC1
**Abdominal Circumference**		GA14AC1	GA20AC1	GA21AC1	GA21AC2	GA21AC3	GA36AC1
**Femur Length**		GA14FL1	GA20FL1	GA21FL1	GA21FL2	GA21FL3	GA36FL1
**Estimated Fetal Weight**		GA14EFW1	GA20EFW1	GA21EFW1	GA21EFW2	GA21EFW3	GA36EFW1

Note:
Gestational age (W/D: weeks/days), crown-rump length (CRL) (mm) and nuchal translucency (NT) (mm) taken once during the week 11 - week 13 (GA11) are denoted by GA11W1, GA11D1, GA11CRL1 and GA11NT1. Gestational age (W/D: weeks/days), biparietal diameter (BPD) (mm), head circumference (HC) (mm), abdominal circumference (AC) (mm), femur length (FL) (mm) and estimated fetal weight (EFW) (g) taken once during the week 14 - week 19 (GA14), once in the week 20 (GA20), three times during the week 21 - week 35 (GA21) and once in the week 36 or later (GA36) are denoted by: GA14W1, GA14D1, GA20W1, GA20D1, GA21W1, GA21D1, GA21W2, GA21D2, GA21W3, GA21D3, GA36W1 and GA36D1 (gestational age); GA14BPD1, GA20BPD1, GA21BPD1, GA21BPD2, GA21BPD3 and GA36BPD1 (biparietal diameter); GA14HC1, GA20HC1, GA21HC1, GA21HC2, GA21HC3 and GA36HC1 (head circumference); GA14AC1, GA20AC1, GA21AC1, GA21AC2, GA21AC3 and GA36AC1 (abdominal circumference); GA14FL1, GA20FL1, GA21FL1, GA21FL2, GA21FL3 and GA36FL1 (femur length); GA14EFW1, GA20EFW1, GA21EFW1, GA21EFW2, GA21EFW3 and GA36EFW1 (estimated fetal weight).

Table S2-1 Random Forest Variable Importance (VI) and Regression Coefficient from Run 1: All Variables (Dependent Variable: Newborn's Weight [g])

**Variable**	**Random Forest**		**Linear Regression**	
	VI Value	*VI Rank*	*Coefficient*	*P-Value*
Gestational Age - Delivery (Weeks)	81437955	1	*170.2000	0.0000
GA36EFW1 (g)	59566065	2	*0.3300	0.0000
GA36AC1 (mm)	40359557	3	*3.3950	0.0055
Maternal Weight at Delivery Time (kg)	12600886	4	-2.9740	0.8606
GA36BPD1 (mm)	11536549	5	3.5670	0.2456
Maternal BMI at Delivery Time	9183122	6	25.9200	0.5537
Neonatal Intensive Care Unit Hospitalization	8666421	7	*-45.8000	0.0265
GA36FL1 (mm)	8167498	8	0.8415	0.8153
GA11CRL1 (mm)	7670171	9	-0.1804	0.8833
GA21AC1 (mm)	7394983	10	1.7720	0.0624
GA21BPD2 (mm)	7337688	11	3.0080	0.3310
Maternal Pregestational BMI	6918490	12	-12.7300	0.7878
GA21AC2 (mm)	6435064	13	1.2240	0.1581
GA21AC3 (mm)	6019105	14	*3.7860	0.0039
GA36HC1 (mm)	5840288	15	-0.1518	0.8389
GA21BPD1 (mm)	5804584	16	3.5090	0.2108
GA20AC1 (mm)	5174051	17	0.2379	0.3931
GA20EFW1 (g)	5113174	18	-0.2566	0.1524
Maternal Pregestational Weight (kg)	5066332	19	1.8500	0.9193
GA21EFW1 (g)	4834716	20	*0.2289	0.0472
GA21FL2 (mm)	4749409	21	-3.1040	0.4481
GA20HC1 (mm)	4713837	22	-0.0199	0.9811
GA21HC2 (mm)	4646094	23	-0.6821	0.3531
Apgar Score in 1 Minute After Delivery	4645375	24	0.4369	0.9574
Apgar Score in 5 Minutes After Delivery	4632233	25	2.7750	0.8193
GA21EFW3 (g)	4602151	26	0.0566	0.5850
Maternal Height (cm)	4570952	27	8.3310	0.3864
GA14BPD1 (mm)	4472051	28	*6.8380	0.0270
Maternal Age	4345422	29	-2.3960	0.1433
GA20BPD1 (mm)	4297216	30	-6.3500	0.0544
GA21EFW2 (g)	4291562	31	*0.2961	0.0005
GA21FL1 (mm)	4270426	32	-4.0100	0.2977
GA21HC1 (mm)	4261178	33	-0.3190	0.3783
GA20FL1 (mm)	4222331	34	*9.9590	0.0214
GA14FL1 (mm)	4186594	35	*-9.4160	0.0486
GA11NT1 (mm)	4115745	36	-2.3880	0.6990
GA14AC1 (mm)	3751675	37	-0.6944	0.5587
GA14HC1 (mm)	3564844	38	0.9472	0.3295
GA21D2	3276785	39	*-15.1600	0.0002
GA36W1	3209062	40	*-90.3100	0.0000
GA14EFW1 (g)	3195941	41	0.2681	0.6498
GA21FL3 (mm)	3148541	42	3.1280	0.5050
GA21BPD3 (mm)	3037689	43	-4.2120	0.2591
Gestational Age - Delivery (Days)	2509973	44	*26.3600	0.0000
GA20D1	2486914	45	-0.7006	0.8328
GA21W2	2448127	46	*-68.6300	0.0000
GA21D1	2403058	47	-4.9800	0.2280
GA14D1	2332492	48	1.0580	0.8048

GA21W3	2210537	49	*-48.2000	0.0000
GA11W1	2101369	50	8.0970	0.6390
GA36D1	2070967	51	*-10.4800	0.0046
GA11D1	1873568	52	2.4760	0.5382
GA21HC3 (mm)	1870408	53	-0.3961	0.6457
GA21D3	1695039	54	-5.1410	0.2215
Newborn's Sex - Female	1690467	55	*-72.8900	0.0000
GA21W1	1672527	56	*-42.2900	0.0002
Number of Ultrasound Equipment Types	1266183	57	-1.0670	0.6962
Caesarean Delivery	1154290	58	*83.4100	0.0000
Maternal Abortions	1112585	59	-5.1360	0.5099
Children Alive	1096311	60	*56.7900	0.0027
Maternal Preterm Births	1093502	61	-36.4600	0.0895
GA14W1	966830	62	-2.5850	0.8723
Maternal Term Births	792117	63	-13.2700	0.4956
GA20W1	0	64		

Notes

**:*	P-Value < 0.05
AC:	Abdominal Circumference (mm)
BPD:	Biparietal Diameter (mm)
CRL:	Crown-Rump Length (mm)
EFW:	Estimated Fetal Weight (g)
FL:	Femur Length (mm)
HC:	Head Circumference (mm)
NT:	Nuchal Translucency (mm)
GA11:	Gestational Age, Week 11 - Week 13
GA14:	Gestational Age, Week 14 - Week 19
GA20:	Gestational Age, Week 20
GA21:	Gestational Age, Week 21 - Week 35
GA36:	Gestational Age, Week 36 or Later
W/D:	Gestational Age - Weeks/Days

Table S2-2 Random Forest Variable Importance (VI) and Regression Coefficient from Run 1: All Variables (Dependent Variable: Benn Index: Newborn's Weight/Height)

**Variable**	**Random Forest**		**Linear Regression**	
	VI Value	*VI Rank*	*Coefficient*	*P-Value*
Gestational Age - Delivery (Weeks)	213	1	*0.2775	0.0000
GA36EFW1 (g)	162	2	*0.0005	0.0026
GA36AC1 (mm)	138	3	*0.0070	0.0050
Maternal Weight at Delivery Time (kg)	38	4	0.0093	0.7880
Maternal BMI at Delivery Time	37	5	0.0004	0.9967
GA36BPD1 (mm)	36	6	0.0072	0.2503
GA21AC1 (mm)	32	7	0.0033	0.0855
GA11CRL1 (mm)	32	8	-0.0002	0.9216
GA21BPD2 (mm)	31	9	0.0041	0.5133
GA21AC2 (mm)	27	10	0.0014	0.4243
GA36FL1 (mm)	23	11	0.0039	0.5915
Maternal Pregestational BMI	22	12	-0.0324	0.7362
GA21EFW2 (g)	22	13	*0.0007	0.0001
Maternal Age	22	14	-0.0049	0.1416
GA21AC3 (mm)	21	15	*0.0061	0.0218
GA21EFW1 (g)	20	16	*0.0006	0.0166
GA21HC2 (mm)	20	17	-0.0010	0.5015
Neonatal Intensive Care Unit Hospitalization	20	18	-0.0460	0.2719
GA21BPD1 (mm)	19	19	0.0014	0.8032
GA36HC1 (mm)	18	20	-0.0016	0.2824
GA20AC1 (mm)	18	21	0.0007	0.2050
Maternal Pregestational Weight (kg)	18	22	0.0095	0.7981
GA14FL1 (mm)	18	23	-0.0176	0.0694
Apgar Score in 1 Minute After Delivery	17	24	0.0016	0.9248
GA21FL1 (mm)	17	25	*-0.0188	0.0166
GA21EFW3 (g)	17	26	0.0001	0.5429
GA21HC1 (mm)	17	27	-0.0008	0.2818
GA20EFW1 (g)	17	28	-0.0006	0.0912
Maternal Height (cm)	17	29	-0.0026	0.8923
GA21FL2 (mm)	16	30	-0.0125	0.1315
GA20BPD1 (mm)	16	31	-0.0076	0.2596
GA14BPD1 (mm)	16	32	*0.0145	0.0214
GA20HC1 (mm)	15	33	-0.0004	0.8220
GA11W1	14	34	0.0061	0.8621
GA20FL1 (mm)	14	35	0.0134	0.1265
GA11NT1 (mm)	13	36	-0.0048	0.7014
Apgar Score in 5 Minutes After Delivery	13	37	0.0130	0.5985
GA14AC1 (mm)	13	38	-0.0015	0.5294
GA14HC1 (mm)	12	39	0.0002	0.9016
GA14EFW1 (g)	12	40	0.0004	0.7231
GA21BPD3 (mm)	12	41	-0.0060	0.4312
GA21D2	11	42	*-0.0258	0.0017
GA21FL3 (mm)	11	43	0.0033	0.7297
GA21W2	9	44	*-0.1161	0.0000
GA36W1	9	45	*-0.1473	0.0000
GA20D1	8	46	-0.0016	0.8136
GA14D1	8	47	0.0077	0.3783
Gestational Age - Delivery (Days)	8	48	*0.0433	0.0000

GA21W3	8	49	*-0.0936	0.0000
GA21D1	8	50	-0.0053	0.5268
GA11D1	7	51	0.0013	0.8756
GA21HC3 (mm)	7	52	0.0009	0.6186
GA36D1	6	53	*-0.0201	0.0074
GA21D3	6	54	-0.0058	0.4996
GA21W1	5	55	*-0.0508	0.0249
Newborn's Sex - Female	5	56	*-0.1179	0.0000
Maternal Abortions	4	57	0.0024	0.8772
Caesarean Delivery	4	58	*0.1594	0.0000
Children Alive	4	59	*0.0883	0.0216
Number of Ultrasound Equipment Types	4	60	0.0026	0.6399
GA14W1	4	61	0.0127	0.6964
Maternal Term Births	4	62	-0.0294	0.4582
Maternal Preterm Births	4	63	-0.0448	0.3044
GA20W1	0	64		

Notes

**:*	P-Value < 0.05
AC:	Abdominal Circumference (mm)
BPD:	Biparietal Diameter (mm)
CRL:	Crown-Rump Length (mm)
EFW:	Estimated Fetal Weight (g)
FL:	Femur Length (mm)
HC:	Head Circumference (mm)
NT:	Nuchal Translucency (mm)
GA11:	Gestational Age, Week 11 - Week 13
GA14:	Gestational Age, Week 14 - Week 19
GA20:	Gestational Age, Week 20
GA21:	Gestational Age, Week 21 - Week 35
GA36:	Gestational Age, Week 36 or Later
W/D:	Gestational Age - Weeks/Days

Table S2-3 Random Forest Variable Importance (VI) and Regression Coefficient from Run 1: All Variables (Dependent Variable: Ponderal Index: Newborn's Weight/Height^3)

**Variable**	**Random Forest**		**Linear Regression**	
	VI Value	*VI Rank*	*Coefficient*	*P-Value*
GA21AC1 (mm)	1804	1	0.0061	0.6314
GA36AC1 (mm)	1598	2	0.0296	0.0697
Gestational Age - Delivery (Weeks)	1417	3	*0.3250	0.0000
GA21BPD2 (mm)	1230	4	0.0145	0.7260
Maternal BMI at Delivery Time	1205	5	-0.1966	0.7364
GA36EFW1 (g)	1196	6	0.0006	0.5464
GA21AC2 (mm)	1068	7	-0.0012	0.9168
Maternal Age	1054	8	-0.0311	0.1542
GA11CRL1 (mm)	868	9	0.0031	0.8499
GA21EFW2 (g)	808	10	*0.0031	0.0064
Maternal Pregestational BMI	728	11	-0.2214	0.7256
Apgar Score in 5 Minutes After Delivery	727	12	-0.1616	0.3187
GA21EFW1 (g)	719	13	*0.0032	0.0373
GA36BPD1 (mm)	672	14	0.0163	0.6903
GA21HC2 (mm)	671	15	-0.0012	0.9037
Maternal Weight at Delivery Time (kg)	662	16	0.0748	0.7408
GA21FL2 (mm)	657	17	*-0.1148	0.0356
GA20FL1 (mm)	572	18	-0.0284	0.6232
GA20BPD1 (mm)	559	19	0.0174	0.6932
GA20EFW1 (g)	558	20	-0.0032	0.1774
Maternal Height (cm)	545	21	-0.1067	0.4059
GA36FL1 (mm)	541	22	0.0587	0.2220
GA11NT1 (mm)	530	23	-0.0205	0.8034
GA21BPD1 (mm)	527	24	-0.0365	0.3298
GA14BPD1 (mm)	520	25	0.0536	0.1934
GA21FL1 (mm)	512	26	*-0.1944	0.0002
GA20AC1 (mm)	489	27	0.0057	0.1269
GA20HC1 (mm)	472	28	-0.0023	0.8358
Maternal Pregestational Weight (kg)	462	29	0.0990	0.6846
GA21AC3 (mm)	462	30	0.0126	0.4697
GA14HC1 (mm)	444	31	-0.0049	0.7033
GA14FL1 (mm)	437	32	-0.0740	0.2455
Apgar Score in 1 Minute After Delivery	431	33	0.0915	0.4015
GA11W1	426	34	-0.0138	0.9522
GA21FL3 (mm)	407	35	0.0151	0.8092
GA21HC1 (mm)	398	36	-0.0063	0.1907
GA21BPD3 (mm)	375	37	-0.0044	0.9290
GA21EFW3 (g)	374	38	0.0005	0.7374
GA14AC1 (mm)	370	39	-0.0016	0.9180
GA36HC1 (mm)	355	40	-0.0150	0.1332
GA14EFW1 (g)	317	41	0.0003	0.9701
GA21D2	316	42	-0.0900	0.0951
GA21HC3 (mm)	308	43	0.0209	0.0686
GA21W1	240	44	0.1859	0.2112
GA21W2	233	45	-0.2402	0.0631
GA11D1	232	46	-0.0273	0.6116
GA14D1	219	47	0.0539	0.3455
GA20D1	218	48	-0.0015	0.9730

Gestational Age - Delivery (Days)	217	49	0.0555	0.1816
GA21D1	209	50	0.0506	0.3582
GA21D3	186	51	0.0240	0.6686
GA36D1	171	52	-0.0703	0.1538
Number of Ultrasound Equipment Types	150	53	0.0532	0.1448
GA21W3	149	*54	-0.3869	0.0012
Maternal Term Births	144	55	-0.1025	0.6934
Neonatal Intensive Care Unit Hospitalization	143	56	0.0954	0.7289
Children Alive	137	57	0.0747	0.7672
Maternal Abortions	137	58	0.0837	0.4208
GA36W1	106	*59	-0.3329	0.0153
Caesarean Delivery	99	*60	0.4253	0.0158
Maternal Preterm Births	99	61	-0.1428	0.6180
GA14W1	87	62	0.0974	0.6498
Newborn's Sex - Female	62	63	-0.2758	0.1077
GA20W1	0	64		

Notes

**:*	P-Value < 0.05
AC:	Abdominal Circumference (mm)
BPD:	Biparietal Diameter (mm)
CRL:	Crown-Rump Length (mm)
EFW:	Estimated Fetal Weight (g)
FL:	Femur Length (mm)
HC:	Head Circumference (mm)
NT:	Nuchal Translucency (mm)
GA11:	Gestational Age, Week 11 - Week 13
GA14:	Gestational Age, Week 14 - Week 19
GA20:	Gestational Age, Week 20
GA21:	Gestational Age, Week 21 - Week 35
GA36:	Gestational Age, Week 36 or Later
W/D:	Gestational Age - Weeks/Days

Figure S1-1 Random Forest Variable Importance Values of Top 20 Predictors for Newborn's Weight

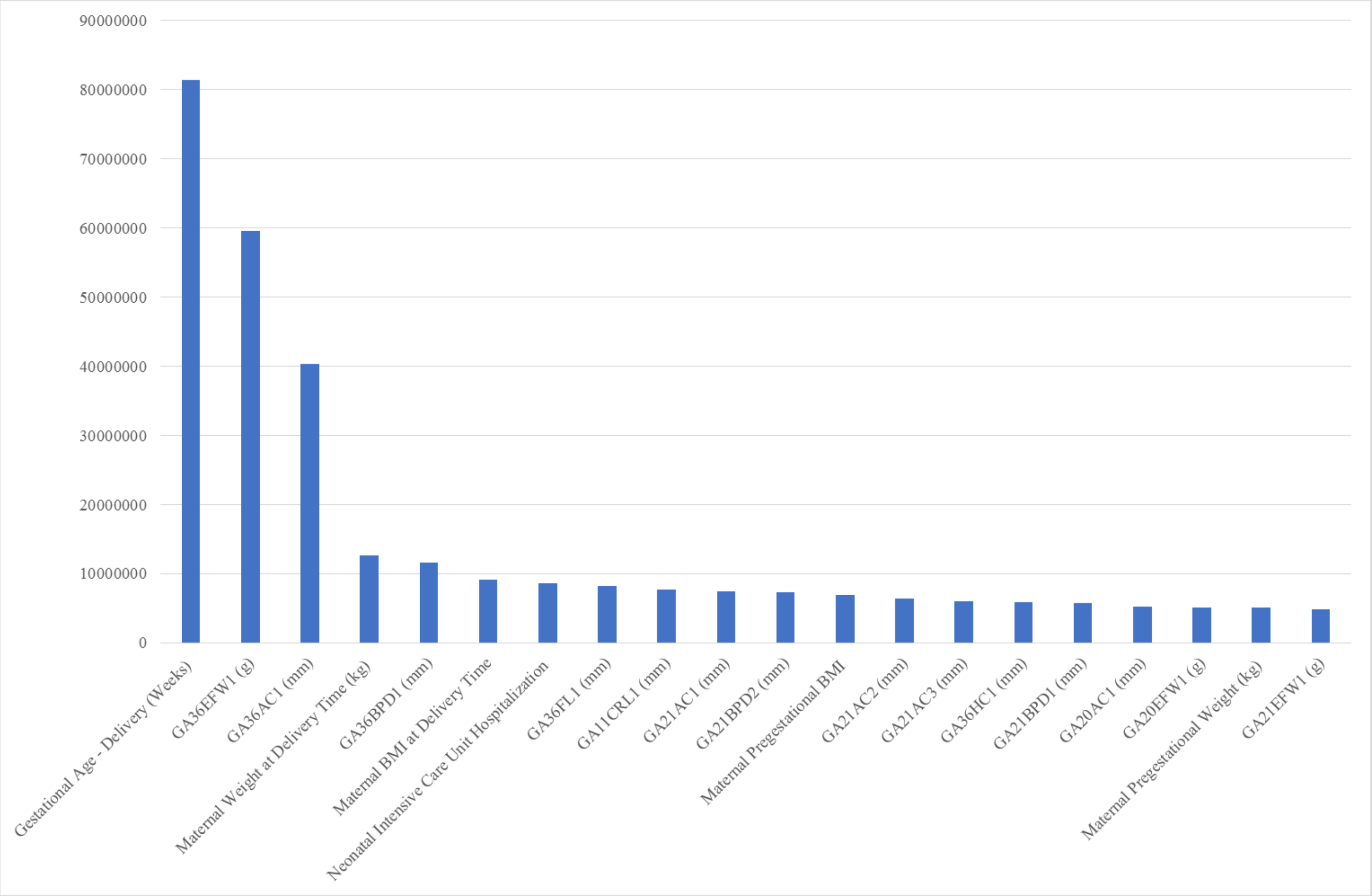

Figure S1-2 Random Forest Variable Importance Values of Top 20 Predictors for Newborn's Weight/Height

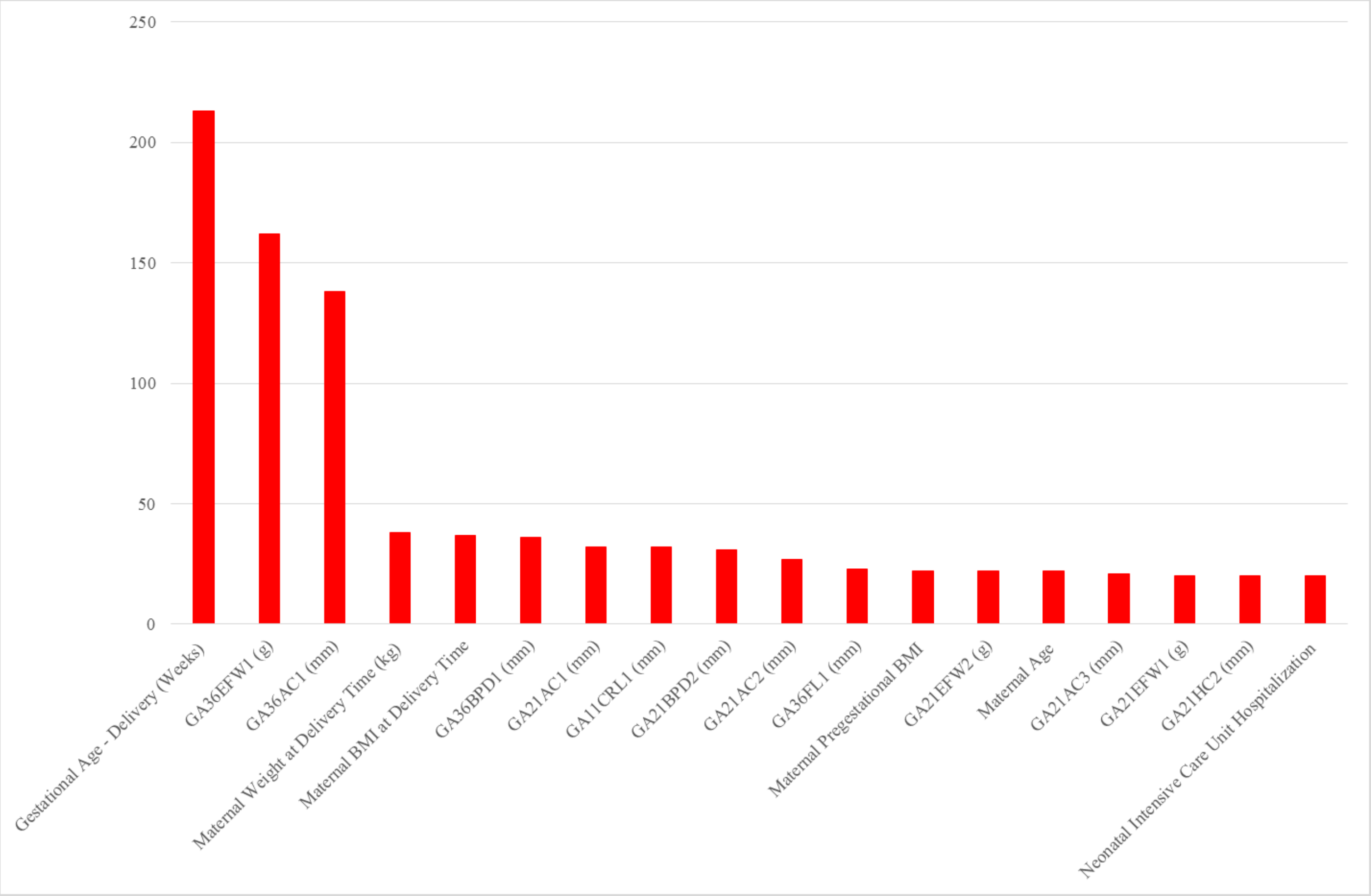

Figure S1-3 Random Forest Variable Importance Values of Top 20 Predictors for Newborn's Weight/Height[3]

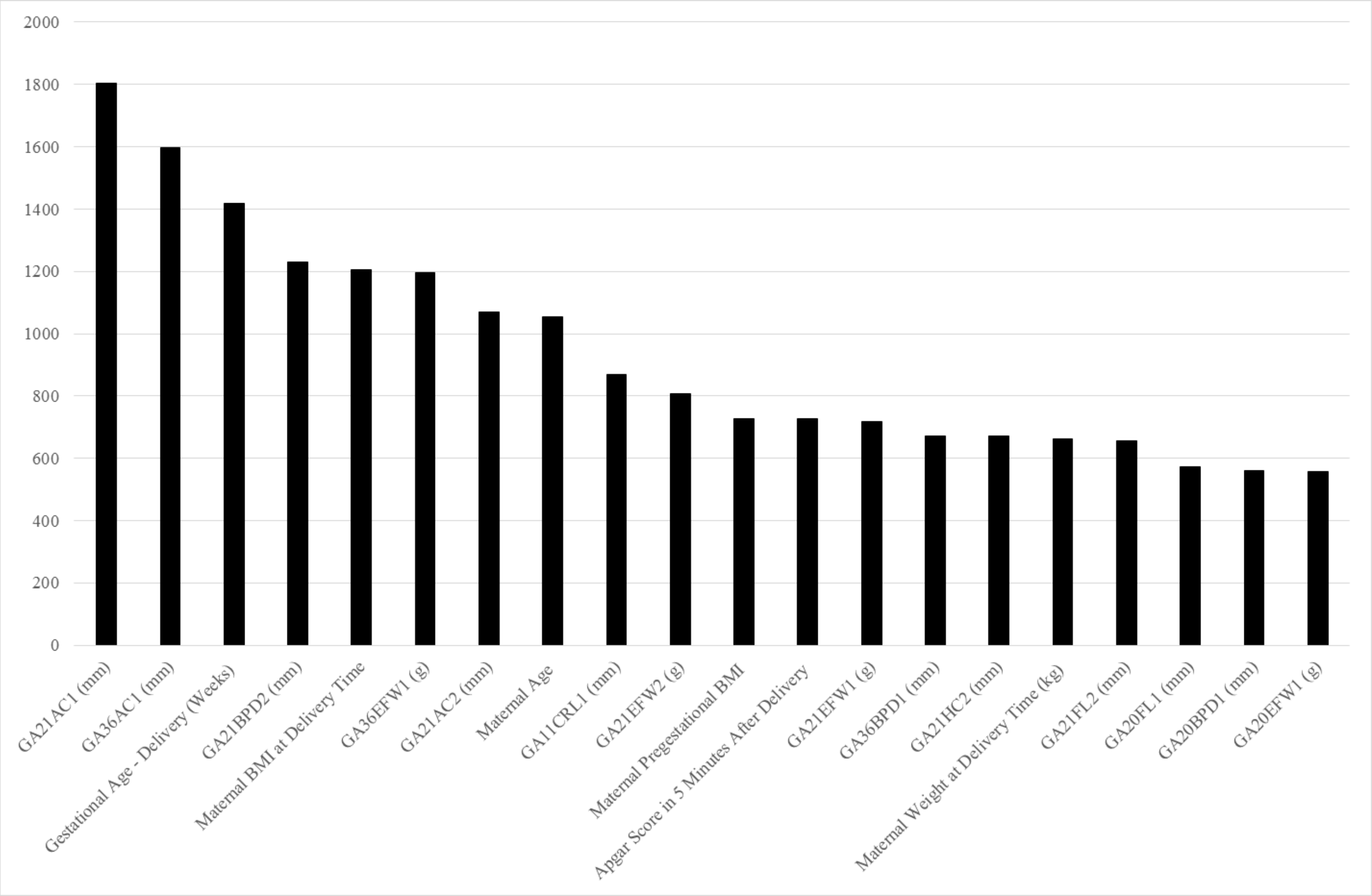

Supplement: Supplementary file 1 [file diagnostics-11-01280-s001.zip › diagnostics-1288566-supplementary.pdf]
